# Single Port Robotic Pyeloplasty: early single-center experience

**DOI:** 10.1590/S1677-5538.IBJU.2023.0406

**Published:** 2024-02-07

**Authors:** Francesco Ditonno, Antonio Franco, Celeste Manfredi, Alexander K. Chow, Srinivas Vourganti, Edward E. Cherullo, Riccardo Autorino

**Affiliations:** 1 Rush University Medical Center Department of Urology Chicago IL USA Department of Urology, Rush University Medical Center. Chicago, IL, USA;; 2 University of Verona Department of Urology Verona Italy Department of Urology, University of Verona. Verona, Italy;; 3 La Sapienza University Sant'Andrea Hospital Department of Urology Rome Italy Department of Urology, Sant'Andrea Hospital, La Sapienza University, Rome, Italy;; 4 "Luigi Vanvitelli" University Department of Woman, Child and General and Specialized Surgery Urology Unit Naples Italy Urology Unit, Department of Woman, Child and General and Specialized Surgery, "Luigi Vanvitelli" University. Naples, Italy

**Keywords:** Robotic Surgical Procedures, Cakut [Supplementary Concept], Hydronephrosis

## Abstract

**Purpose:**

Ureteropelvic junction obstruction (UPJO) is a prevalent cause of hydronephrosis, especially in young patients. The treatment paradigm for this condition has shifted from open to minimally invasive pyeloplasty. In the present study we describe our initial single centre experience with single port (SP) robot-assisted pyeloplasty (RAP) via periumbilical incision.

**Material and methods:**

With the patient in a 60-degree left flank position, the SP system is docked with the Access port (Intuitive Surgical, Sunnyvale, CA, US) placed in a periumbilical 3 cm incision. Robotic instruments are deployed as follows: camera at 12 o'clock, bipolar grasper at 9 o'clock, scissors at 3 o'clock and Cadiere at 6 o'clock. After isolation and identification of the ureter and the ureteropelvic junction (UPJ), the ureter is transected at this level and then spatulated. Anastomosis is carried out by two hemicontinuous running sutures, over a JJ stent.

**Results:**

Between 2021 and 2023, a total of 8 SP RAP have been performed at our institution, with a median (interquartile range, IQR) of 23 years (20.5-36.5). Intraoperative outcomes showed a median (IQR) OT of 210.5 minutes (190-240.5) and a median (IQR) estimated blood loss (EBL) of 50 mL (22.5-50). No postoperative complications were encountered, with a median (IQR) length of stay (LOS) of 31 hours (28.5-34).

**Conclusion:**

In the present study we evaluated the feasibility and safety of SP RAP. The observed outcomes and potential benefits, combined with the adaptability of the SP platform, hold promising implications for the application of SP system in pyeloplasty treatment.

## INTRODUCTION

Ureteropelvic junction obstruction (UPJO) is a prevalent cause of hydronephrosis, characterized by flank pain and urinary tract infections (UTIs) as its most common clinical presentation. The widespread adoption of laparoscopic and robotic approaches has transformed the treatment paradigm for this condition, shifting from an open approach to a minimally invasive surgery, particularly in adults (1). Despite the clear advantages of laparoscopy over the open approach, including shorter hospital stays and lower rates of perioperative complications, it is associated with several drawbacks, such as a longer learning curve, advanced skill requirements, ergonomic limitations and increased operative time (OT) (2). When laparo-endoscopic single site (LESS) pyeloplasty was described, to reduce the need for analgesics while enhancing recovery time, it failed to demonstrate any advantage over conventional laparoscopy (3). On the contrary, the most important drawback of LESS pyeloplasty was represented by the ergonomics of the procedure, especially when a precise dissection and intracorporeal reconstruction is needed (4). The advent of robotic surgery, with its three-dimensional vision and tremor filtration capabilities, has simplified this procedure, overcame the limitations of conventional laparoscopy, and enabled easier dissection and suturing (5). Therefore, the number of pyeloplasty performed with a robot-assisted approach had a 10-fold increase between 2002 and 2008 (1).

With the aim of minimizing skin incision and working in smaller spaces while retaining the advantages of robotic instruments, the Da Vinci Single Port (SP) system (Intuitive Surgical, Sunnyvale, CA™) has gained popularity among surgeons trained in robotic procedures and is currently adopted for many urologic procedures. Preliminary data with this approach reveal encouraging results, demonstrating perioperative and intermediate-term outcomes comparable to standard MP robotic-assisted pyeloplasty (6, 7). Nonetheless, up to date, the available literature on SP pyeloplasty in adults is limited to retrospective studies, consisting mainly of initial clinical experiences and small case series.

In the present study we share our initial single centre experience encompassing all consecutive patients who underwent SP robot-assisted pyeloplasty (RAP) via an umbilical incision between November 2021 and July 2023.

## SURGICAL TECHNIQUE

At our Rush University Medical Center, IRB criteria was met, and approval granted (protocol no. 22111003) prior to surgery and data collection.

SP RAP is performed following the Anderson- Hynes dismembered pyeloplasty technique (8).

The patient is placed in a 60-degree left flank position, under general anaesthetic. Through a periumbilical 3 cm incision, peritoneum is entered under direct vision and the Access port (Intuitive Surgical, Sunnyvale, CA, US™) introduced, with an 8mm Airseal port to allow pneumoperitoneum, set at 10-12 mmHg. The SP robot is docked with camera at 12 o'clock, bipolar grasper at 9 o'clock, scissors at 3 o'clock and Cadiere at 6 o'clock. Suction is carried out using the remotely operated suction irrigation system (ROSI, Vascular Technology Inc, Nashua, NH), directly manipulated by the surgeon using robotic instruments.

**Figure 1:**
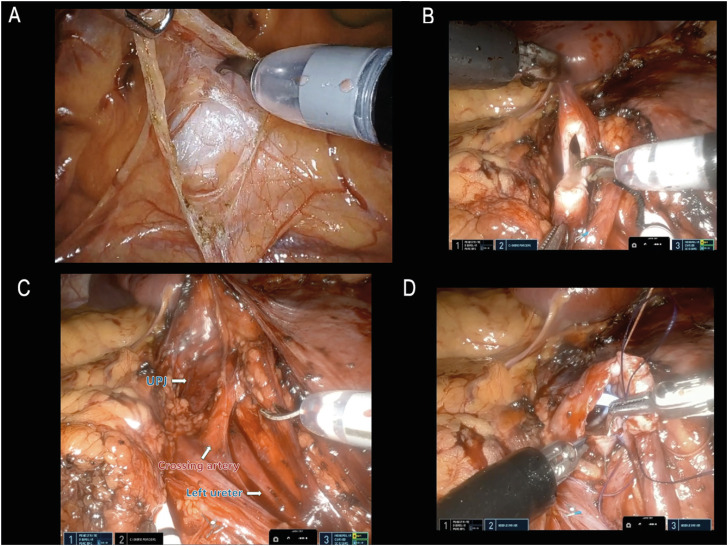
Step by step surgical procedure (A: left mesenteric incision for direct access to ureteropelvic junction; B: Ureter transection at ureteropelvic junction level; C: a crossing polar renal artery clearly abutting the ureter at the level of the lower pole; D: anterior aspect of the ureteropelvic anastomosis after JJ stent placement).

After entering peritoneal cavity, the descending colon is reflected medially using cautery and scissors. Dissection is carried out until psoas muscle and ureter are identified and traced upwards. As an alternative, a transmesenteric approach may be employed for left pyeloplasty, allowing for direct access to the renal pelvis (Figure-1A). The ureter is then transected at level of the UPJ and then spatulated (Figure-1B). When encountering a transecting vessel, the UPJ is transposed and reconstructed anteriorly to it (Figure-1C). At this point, the renal pelvis can be reduced, according to its grade of dilatation. In our index case we did not perform this step, given its normal size. Anastomosis is carried out by two hemicontinuous 4-0 RB-1 Vicryl running sutures. Through the Access port and before completing the anterior part of the UPJ anastomosis, a JJ stent is introduced into the ureter over a guide, with the proximal coil placed in the renal pelvis (Figure-1D). Once the two sides of the anastomosis are completed, the sutures are tied together at its vertex. Drain was omitted in all cases. The fascia is closed with interrupted figure-of-eight, skin edges with subcuticular and Dermabond applied. Step-by-step procedure is visually displayed in the Supplementary video (Supplementary material).

Between 2021 and 2023, a total of 8 SP RAP have been performed at our institution, with a median (interquartile range, IQR) of 23 years (20.5-36.5). Most common presentations were flank pain (5, 62.5%), and only one case of previous UTI was observed. All the patients had mild to moderate hydronephrosis at preoperative imaging, with a median (IQR) preoperative serum creatinine (SCr) of 1.06 mg/dL (0.96-1.18) and a preoperative estimated glomerular filtration rate (eGFR) of 86 mL/min/1.73 m2 (78-97). Intraoperative outcomes showed a median (IQR) OT of 210.5 minutes (190-240.5) and a median (IQR) estimated blood loss (EBL) of 50 mL (22.5-50). No postoperative complications were encountered, with a median (IQR) length of stay (LOS) of 31 hours (28.5-34). Median (IQR) DVPRS (Defense and Veterans Pain Rating Scale) score at discharge was 4 (2.5-6). Postoperative opioids consumption has been quantified in morphine milligram equivalent (MME). Median (IQR) daily MME amounted to 20 mg (range: 10-30), whereas the total postoperative MME was 55 mg (17.5-87.5). The median (IQR) time to stent removal was 42 days (30-46.5). At early follow-up, postoperative renal function resulted in a SCr of 1.07 mg/dL (0.89-1.11), with an eGFR of 93 mL/min/1.73 m2 (80.5-113.5) (Table-1).

**Table 1:** Patients baseline features and perioperative outcomes.

SP RAP (n=8)		
Preoperative features		Median (IQR)
Age (years)		23 (20.5-36.5)
**Presentation**		
	Flank pain (n, %)	5/8 (62.5%)
	UTI (n, %)	2/8 (20%)
Hydronephrosis (n, %)		8/8 (100%)
Preop SCr (mg/dL)		1.06 (0.96-1.18)
Preop eGFR (mL/min/1.73 m^2^)		86 (78-97)
**Perioperative outcomes**		**Median (IQR)**
OT (min)		210.5 (190-240.5)
EBL (mL)		50 (22.5-50)
LOS (hours)		31 (28.5-34)
DVPRS score		4 (2.5-6)
Complications (n, %)		0/8 (0%)
Time to stent removal (days)		42 (30-46.5)
Postop SCr (mg/dL)		1.07 (0.89-1.11)
Postop eGFR (mL/min/1.73 m^2^)		93 (80.5-113.5)

Abbreviations: SP RAP (single port robot-assisted pyeloplasty); UTI (Urinary tract infection); SCr (serum creatinine); eGFR (estimated glomerular filtration rate); OT (operative time); EBL (estimated blood loss); LOS (length of stay); DVPRS (Defense and Veterans Pain Rating Scale).

## COMMENTS

In our initial experience SP RAP demonstrated its safety and feasibility for the treatment of UPJO in adults, as already suggested by evidence available in the current literature.

For what concerns intraoperative outcomes, Bekcsac et al. reported an OT of 159 min, with 22.27 mL of EBL, with no significant difference compared to multi-port (MP) approach (7). Interestingly, in a larger series recently published by Harrison et al. the OT was significantly longer for SP RAP, compared to MP RAP (128 min vs 88 min, p=.04), with no difference in terms of EBL (6). In our initial experience, we observed a longer OT (233 minutes), possibly attributable to the learning curve that surgeons involved in the procedure are experiencing. Although a specific learning curve is required when approaching the SP system, it is considerably shorter for surgeons who already have experience in robotic surgery. Consequently, outcomes obtained with the SP approach are readily comparable to those obtained with the MP approach. An extremely low EBL was observed in this series, as it was in previous studies (6, 7, 9), confirming the safety of this procedure. Moreover, no postoperative complications were registered, and all the patients were discharged on postoperative day one, following an internal protocol, with a median LOS of 31 hours and a median DVPRS score of 4. A recent systematic review and meta-analysis by Gu et al. reported no major complications following SP RAP, with no significant difference between SP-RP and MP-RP in terms of complications rate, thus corroborating our results (9). An additional potential benefit associated with SP approach may be related to the shorter LOS. Indeed, Beksac et al. provided evidence of significantly reduced LOS in patients undergoing SP RAP (12.18 hours), compared to MP RAP (42.66 hours, p<.001) (7). This finding was further confirmed by Gu et al. in their meta-analysis, which confirmed the advantage of the SP platform in terms of significantly shorter LOS for patients undergoing pyeloplasty (9). Thus, SP technique is highly appealing in the context of inpatient care, potentially leading to cost advantages for both patients and the healthcare system.

Several features of the SP platform make it well-suited for pyeloplasty, such as the need of a small working area and the possible application in bilateral UPJO. The added value of SP platform lies in its pliability, showed by its capability to perform various types of accesses and docking, enabling surgeons to adapt their approach to specific patient anatomy and procedural requirements. Therefore, in addition to a transperitoneal approach, the same procedure could be carried out in a retroperitoneal fashion, with patient placed in flank or supine position, while retaining the advantages reported in the present study. The feasibility of a supine approach for renal surgery has been demonstrated by Pellegrino et al. in a pilot study, wherein they applied a retroperitoneal approach for upper urinary tract pathologies with the patient in supine position using the da Vinci SP robot (10). The adoption of this approach for SP RAP could extend to this procedure the benefits associated with retroperitoneal surgery, including enhanced control of hilar structures, reduced operative times and hospital stays, and decreased post-operative discomfort and pain. A reduction in postoperative pain may lead to a decrease in opioid usage, which constitutes the most employed analgesic for postoperative pain in the United States. Similarly, opioid dependence and their excessive utilization following urologic surgery pose a significant public health concern, particularly among younger patients with extended hospital stays (7). The adoption of a SP approach led to an opioid use of 20 mg of MME/daily, and 55 mg of MME in the whole postoperative course.

Among other factors, the potential for improved postoperative cosmesis is highly regarded, considering that young or paediatric patients are frequently involved in this procedure. Various incision sites for SP RAP have been documented in the literature, all yielding satisfactory outcomes. We believe the umbilical incision can achieve a favourable cosmesis results, as the scar remains completely concealed within the umbilicus after healing, and this can be an added benefit in the young adult population. Beckas et al. proposed a mini-Pfennesteil incision with the aim of improving cosmesis and reducing postoperative pain (7). While this may not appear to be the primary determinant when selecting one approach over another, this aspect gains particular significance when considering the relatively young age of patients with UPJO that makes cosmesis potentially of high importance for this specific subgroup of patients.

Our study is not devoid of limitations, in particular the small sample size and the single-center experience which may hamper the validity and generalizability of our results. Furthermore, our study lacks long-term follow-up data, thus no conclusion about long-term functional results can be drawn. Nevertheless, we documented the feasibility and safety of SP RAP, confirming the emerging evidence on this novel approach, and further supporting the use of SP RAP for the treatment of UPJO as an appealing alternative to MP RAP.

## CONCLUSIONS

The present study adds to the expanding field of SP RAP for UPJO in young adults. The observed outcomes and potential benefits, combined with the adaptability of the SP platform, hold promising implications for the application of SP system in pyeloplasty treatment. Further investigations in larger patient cohorts and longitudinal studies are needed to further support the role of SP RAP and confirm its non-inferiority compared to MP RAP, considering its potential advantages in terms of cosmesis, opioid use, and postoperative pain.

## DECLARATIONS

Data Availability. The data sets generated during and/or analysed during the current study are available from the corresponding author on reasonable request.
